# Visual Sensitivity in Complex Regional Pain Syndrome and Fibromyalgia: An Online Study

**DOI:** 10.1177/03010066211072641

**Published:** 2022-03-03

**Authors:** Antonia F. Ten Brink, Janet H. Bultitude

**Affiliations:** 1555 University of Bath, UK

**Keywords:** complex regional pain syndrome, fibromyalgia, visual allodynia, pattern glare, reversible figures, bistable image

## Abstract

Perceptual anomalies can provide insights into underlying pathologies even when they are not the main symptom of many clinical conditions. Complex regional pain syndrome (CRPS) and fibromyalgia are chronic pain conditions associated with changes in the central nervous system, possibly leading to enhanced visual sensitivity. It is unclear whether this occurs more than for people with other types of pain. We examined visual sensitivity elicited by different stimuli and in daily life, through an online study of people with CRPS (*n* = 57), fibromyalgia (*n* = 74), other pain (*n* = 50), and no pain (*n* = 89). Respondents rated changes in pain, discomfort, or distress from viewing patterns with different spatial frequencies (lower-order visual processing), and reversible figures (bistable images; higher-order visual processing). We assessed visual sensitivity in daily life using the Leiden Visual Sensitivity Scale and Visual Discomfort Scale. Respondents with CRPS or fibromyalgia reported more visual discomfort than pain-related and pain-free controls while viewing striped patterns and a circle, with no effect of spatial frequency. They reported more pain while viewing a nonreversible square, but not reversible figures (Necker Cube, Duck/Rabbit). Finally, they reported more daily visual sensitivity than pain-related and pain-free controls. Suppressing visual cortical activity might benefit people with CRPS or fibromyalgia.

## Introduction

Perceptual anomalies are seen in many clinical conditions, as core features and/or as additional complaints outside the diagnostic criteria. For example, complex regional pain syndrome (CRPS) and fibromyalgia are two, chronic musculoskeletal pain conditions that are associated with increased responses to painful and nonpainful somatosensory stimuli (Littlejohn, 2015; Marinus & van Hilten, 2006). Even the feeling of water or a breeze can cause intense pain in people who suffer from these conditions. These increased responses are absent or less severe in other pain conditions such as arthritis (Palmer et al., 2019), and have been attributed to a hyperactive or over sensitive central nervous system, called central sensitization (Adams & Turk, 2015; Ji & Woolf, 2001; Yunus, 2008). CRPS and fibromyalgia have distinct clinical phenotypes: CRPS involves regional pain (Birklein et al., 2018; Stanton-Hicks et al., 1995), whereas fibromyalgia involves widespread pain (Hawkins, 2013; Häuser et al., 2015; Wolfe et al., 2016). However, they also share many overlapping features (Littlejohn, 2015; Littlejohn & Guymer, 2019; Marinus & van Hilten, 2006), and both syndromes are associated with functional and structural changes in the central nervous system (Henry et al., 2011; Kindler et al., 2011; Littlejohn, 2015; Yunus, 2008).

It is proposed that in both CRPS and fibromyalgia sensory input across multiple systems is amplified by the central nervous system, leading to enhanced sensitivity for *nonsomatic* stimuli as well, such as visual stimuli (de Klaver et al., 2007; Fleming & Volcheck, 2015). Anecdotally, some people with CRPS or fibromyalgia report pain and discomfort when looking at high-contrast images (Dönmez et al., 2012; Ten Brink et al., 2020). Another potential source of visual discomfort in CRPS and fibromyalgia is sensory conflict, including visual conflict (Brun et al., 2019; Cohen et al., 2012; Knudsen & Drummond, 2015; McCabe et al., 2007). Optokinetic stimulation inducing the illusion of movement (Knudsen & Drummond, 2015), or viewing an ambiguous reversible figure (Cohen et al., 2012; Hall et al., 2011), worsens the pain in some people with CRPS. However, there is little understanding of whether people with CRPS or fibromyalgia experience such discomfort to a greater extent than people with other types of chronic pain or pain-free controls. For example, particular patterns of striped lines induce visual distortions along with discomfort, headaches, and nausea in people without pain (Conlon et al., 2001; Wilkins et al., 1984), which is worse in migraine sufferers (Evans & Stevenson, 2008; Harle et al., 2006; Shepherd, 2001). Sensory conflict can also generate unpleasant sensations, such as motion sickness, in people without pain (McCabe, 2005; Warwick-Evans et al., 1998). Furthermore, there is little understanding of what types of images elicit such pain and discomfort in people with CRPS or fibromyalgia, and whether such visual sensitivity is related to discomfort in daily life situations. A dissociation can be made between stimuli that involve mainly lower order visual processing (e.g., striped patterns) or both lower and higher order visual processing (e.g., reversible images). Viewing reversible or bistable images has been related to activity in both low-level visual regions and higher order regions, including frontal areas, although there is discussion on whether the frontal activity is a consequence of transitions between percepts, or the cause (i.e., reflecting the conscious top-down mechanism of voluntary induced switches; de Graaf et al., 2011; Kleinschmidt et al., 2002; Knapen et al., 2011; Lumer, 1998).

There are several possible neural mechanisms underlying visual discomfort. The main hypothesis regards the large hemodynamic response in the visual cortex. Striped patterns give rise to an inefficient and less sparse encoding in the visual cortex, which induces a large hemodynamic response (Hibbard & O’Hare, 2015; O’Hare & Hibbard, 2011; Penacchio & Wilkins, 2015). In general, large hemodynamic responses are associated with discomfort, suggesting that the discomfort is homeostatic and acts to reduce the excessive metabolic demand (Haigh et al., 2015). Indeed, healthy people who report more discomfort glare (i.e., induced by a bright LED) show an increased neuronal response in low-level visual areas than people who report less discomfort glare (Bargary, Furlan, et al., 2015). An alternative hypothesis relates to specific spatial frequencies. Cortical cells responding to specific spatial frequencies lead to increased levels of excitation in a localized area of the visual system, which is associated with unpleasant effects. It has been hypothesized that a failure of inhibitory mechanisms among spatial-frequency channels at a cortical level may allow this effect, although further research is warranted (Conlon et al., 2001). In people with migraine, a hyperexcitable cortex may cause a spread of excitation, leading to neurons firing inappropriately and thereby resulting in stronger perceptual illusions and distortions, and possibly promoting migraine attacks (Wilkins et al., 2007). As central sensitization is thought to play a role in CRPS and fibromyalgia, both mechanisms could underly enhanced visual discomfort after viewing (nonnatural) geometric patterns in these groups. It is likely that such mechanisms are at play in other (pain) conditions as well. For example, people with myalgic encephalomyelitis/chronic fatigue syndrome have an increased vulnerability to pattern-related visual discomfort (Wilson et al., 2015), and people with mild traumatic brain injury more often report photosensitivity (Truong et al., 2014).

The aim of this study was to investigate visual sensitivity for striped patterns (involving lower-order visual processing) and reversible figures (involving higher-order visual processing) in people with CRPS or fibromyalgia. In an online study, we asked respondents who reported as having received a diagnosis of CRPS, fibromyalgia, other pain, or no pain to look at striped patterns and reversible figures that have previously been shown to cause more visual distortions and discomfort in people with than without chronic pain (Cohen et al., 2012; Evans & Stevenson, 2008; Hall et al., 2011; Harle et al., 2006; Marcus & Soso, 1989; Wilson et al., 2015). We hypothesized that respondents who reported as having received a diagnosis of CRPS or fibromyalgia would report more visual distortions and pain, discomfort, and distress when looking at striped patterns and reversible figures compared to respondents with other types of chronic pain or pain-free controls. We expected that this difference would be largest for images that have been previously associated with the greatest distortions. We also hypothesized that respondents who reported as having received a diagnosis of CRPS or fibromyalgia would experience more figure reversals (Cohen et al., 2012; Hall et al., 2011). Finally, we investigated whether respondents who reported as having received a diagnosis of CRPS or fibromyalgia would self-report more light and pattern sensitivity in daily life situations compared to respondents with other types of chronic pain or pain-free controls, and whether this sensitivity was related to pain, discomfort, and distress for the striped patterns and reversable figures.

## Methods

### Online Study

#### Study Distribution and Procedure

We created an online study using Qualtrics software (Qualtrics, 2005). We distributed the link to this study to people with CRPS, fibromyalgia, other types of pain, and pain-free controls who had previously taken part in other research for our research group and who had agreed to be contacted about future studies. Additionally, we distributed the study link to the Community Participant Panel of the Psychology Department of the University of Bath, through patient newsletters and social media groups for a number of pain conditions, via our own social media, and to our friends and relatives. We collected responses from December 2019 to April 2020. The study was automatically closed if respondents opened the survey on a mobile device, did not provide informed consent, were aged below 16 years, or indicated that they had a history of neurological illness/injury or epilepsy. Respondents were offered the chance to enter into a £50 Amazon gift voucher prize draw. The study was designed to take no more than 25 min to complete. The study was approved by the Psychology Research Ethics Committee of the University of Bath (PREC code 19-278). We uploaded a preregistration at the Open Science Framework website https://osf.io/td93k/.

The sections of the study are described in detail below. First, respondents were presented with general information about the study, a CAPTCHA test to block robotic responses, and an informed consent page. Next, respondents were asked for demographic and pain-related information. Then, the study progressed to the striped pattern test section and the reversible figure sections (order counterbalanced). After each striped pattern or reversible figure, respondents were invited to express any thoughts, feelings or sensations that they experienced while looking at the previous image in a free-text box for analysis in a related qualitative study. Then, they filled out the Leiden Visual Sensitivity Scale (L-VISS) and the Visual Discomfort Scale.

#### Demographic and Pain-Related Information

Respondents were asked to indicate their age, sex, and handedness. Respondents were asked whether they experienced pain most of the days for 3 months or more. If this question was answered with “yes,” respondents were asked questions on their pain duration (in years) and the average hours of pain they experienced per day. Using a selection of 10 predefined body parts and a free-text “other” box, respondents with chronic pain were asked to indicate in which area/part of their body they experienced pain in the past week (Supplemental Table S1). All respondents were asked whether they had received a pain-related medical diagnosis, and what this diagnosis was. We predefined 15 pain-related medical diagnoses; including CRPS (we did not dissociate between CRPS I and CRPS II, as many people do not know which type they have) and fibromyalgia (Supplemental Table S2). An “other” option was included with a free-text box for respondents to specify additional diagnoses. Respondents were asked to rate their levels of pain, discomfort, and distress using separate Numerical Pain Rating Scales ranging from 0 (no pain/discomfort/distress) to 10 (worst imaginable pain/discomfort/distress; Karcioglu et al., 2018; Williamson & Hoggart, 2005).

Respondents were asked whether or not they had dyslexia (“Do you have dyslexia?”), as some of the questions on light and pattern sensitivity in daily life regard difficulties with reading, which could be affected by dyslexia (Shepherd et al., 2013; Wilkins et al., 2016). We did not ask whether participants had received a formal diagnosis of dyslexia. Finally, two control questions were included instructing which option had to be selected, in order to assure that respondents had read the questions.

#### Striped Patterns

We used an adapted version of the Pattern Glare Test, an optometric test developed to measure susceptibility to perceptual distortions and discomfort from patterns (Wilkins & Evans, 2001, 2010). The original Pattern Glare Test includes three striped patterns (square-wave gratings) that differ in spatial frequency. The recommended viewing distance is 40 cm, but it is not necessary to precisely control viewing distance and the participant is instructed to view the patterns at their usual reading distance. For ease of reading, we report the cycles per degree (cpd) as though viewed at a distance of 40 cm. Of the three patterns, viewing distance has a large effect on the number of visual distortions only for pattern 3 (stripes of 9.4 cpd at 40 cm, to 14.2 cpd at 60 cm; Conlon et al., 2001; Wilkins et al., 2016). Because we could not control for viewing distance in this online study, we did not use pattern 3. That is, we included only pattern 1 (stripes of 0.3 cpd at 40 cm) and pattern 2 (stripes of 2.3 cpd at 40 cm). Visual distortions are rarely reported for the 0.3 cpd striped pattern in normal populations, and the pattern can be used to account for the person's acquiescence to suggestion in reporting symptoms. The 2.3 cpd striped pattern is likely to induce distortions, and these effects are not influenced by viewing distance. The Pattern Glare Test has been validated in people with migraine (Evans & Stevenson, 2008). We included a filled grey circle as an additional control image, to evaluate potential differences for striped versus nonstriped circles (i.e., both geometric shapes).

Respondents were first presented with an example dot and instructed to maintain their gaze on a similar fixation dot at the center of each subsequent image for 10 s. The three images were shown in a fixed order, as was done in the original Pattern Glare Test. All stimuli had a diameter of 10.4 cm and were presented on a white background. The first stimulus was the filled grey circle (i.e., created in Microsoft PowerPoint), the second stimulus the 0.3 cpd striped pattern (i.e., white and black horizontal stripes of 1.40 cm), the third stimulus the 2.3 cpd striped pattern (i.e., white and black horizontal stripes of 0.15 cm; the striped patterns were provided by the original authors). Stimuli were presented in the center of the horizontal axis of the screen and at the top of the webpage (the exact location on the vertical axis depending on the screen size). Since respondents did the experiment on their own device, we could not control for stimulus luminance. The mouse cursor remained on screen throughout the trial. Next, respondents were asked to answer seven questions about any distortions they perceived (i.e., colors, bending of lines, blurring of lines, shimmer/flicker, fading, shadowy shapes, or other effects); and whether these distortions were mild (scored as 1), moderate (2), or strong (3). The image remained on screen so participants could view the image again if they wished. A total visual distortion score was computed (ranging from 0 to 21). In a next section, where the image was off screen, respondents were asked whether the image increased or decreased their pain, discomfort, and distress on 7-point scales. Each scale ranged from −3 (severe decrease) to +3 (severe increase), 0 being no change. In a previous study (using reversible figures), many people with CRPS looked away from the stimulus images because they were unable to tolerate the discomfort they induced (Hall et al., 2011). Respondents in our study were therefore asked whether they looked away from the image. Respondents who indicated that they did not look at the image for the entire time were asked to indicate the percentage of presented time that they had looked at the image (using a slider). They were also asked to select a reason from the following predefined options: “It caused me too much pain, discomfort or distress,” “I was distracted by something else in the room,” “I was bored,” or “Other (please specify).”

#### Reversible Figures

Reversible figures (or bistable images) are images for which there are two possible interpretations, with the viewer typically switching between each percept in a bistable manner. First, the concept of reversible figures was explained to participants with the example of a face-vase figure, a stimulus that was not used in the task itself. This example was shown with an explicit instruction about the two perspectives in the figure (i.e., a vase or two faces) to make sure participants knew what to expect throughout the task. Next, respondents were instructed to look at each figure and click on the screen with the computer mouse every time they noticed a figure reversal. A sequence of three figures was presented in random order: a reversible Duck/Rabbit figure (a natural image), a reversible Necker Cube, and a nonreversible square (both geometric shapes) that represented the control condition. The choice of stimuli was based on the study of Hall et al. (2011). All stimuli were black, presented on a white background. The Duck/Rabbit figure (10.4 × 8 cm) was retrieved from the “Fliegende Blätter” (1892). The Necker Cube (10.4 × 8.6 cm) and square (10.4 × 10.4 cm) were created in Microsoft PowerPoint. There was no central dot and participant could freely view the image. Stimuli were presented in the center of the horizontal axis of the screen and at the top of the webpage (the exact location on the vertical axis depending on the screen size). Since respondents did the experiment on their own device, we could not control for stimulus luminance. The mouse cursor remained on screen throughout the trial. Each figure was presented for 45 s. After the figure disappeared, respondents were asked whether the figure increased or decreased their pain, discomfort, and distress, on 7-point scales. Each scale ranged from −3 (severe decrease) to +3 (severe increase), 0 being no change. Respondents were asked whether they looked away from the figure, and if so, why they looked away and for how long they had looked at the figure.

#### Light and Pattern Sensitivity in Daily Life

The L-VISS measures the impact of light and pattern sensitivity on daily functioning (Perenboom et al., 2018). Respondents indicate for 9 items whether they experience different forms of pattern sensitivity on a scale from 0 (“not at all”), 1 (“moderately”), 2 (“severely”), to 3 (“very severely”), resulting in a total score of 0–27. The L-VISS has a good to excellent test–retest reliability in people with migraine, and is positively related with the number of visual distortions at the Pattern Glare Test (Perenboom et al., 2018). The L-VISS has a good internal consistency in people with chronic pain (Ten Brink et al., 2021). The questions in the L-VISS relate well to the construct that we aimed to measure: light and pattern sensitivity in daily life. However, the scale is not validated extensively, so we additionally administered the Visual Discomfort Scale.

The Visual Discomfort Scale measures visual sensitivity in daily life situations, with a strong focus on reading (Conlon et al., 1999). Respondents rate 23 different situations on a scale from 0 (“event never occurs”) to 3 (“almost always”), resulting in a total score of 0–69. The questions and response categories were based on the original Visual Discomfort Scale of Conlon et al. (1999). Subdomains that are measured are movement/fading, blur/diplopia, headache/eye soreness, glare, rereading, and slow reading (Borsting et al., 2007). The Visual Discomfort Scale is validated in students (Borsting et al., 2007, 2008), and people with migraine (Cucchiara et al., 2015). The Visual Discomfort Scale has a good internal consistency in people with chronic pain (Ten Brink et al., 2021).

### Statistical Analyses

As several assumptions for parametric testing were violated, we used nonparametric tests for all analyses. The level of alpha was set at 0.05 and we used the Holm-Bonferroni method to correct for multiple post-hoc comparisons (Holm, 1978). Effect sizes were computed with the Pearson correlation coefficient. Pearson correlation coefficients of >0.10 were considered to reflect a small, >0.30 a medium, and >0.50 a large effect (Field, 2005). SPSS version 25 (IBM Corp., 2017) was used to conduct statistical analyses, and R version 3.6.0 (R Core Team, 2021) was used to create the graphs.

#### Respondents

We assigned respondents to one of four groups based on their declared diagnoses or lack thereof: CRPS, fibromyalgia, pain control, or pain-free control. Respondents who reported not to have had pain for 3 months or more were allocated to the pain-free control group. The allocation of respondents to the CRPS or fibromyalgia groups was based on a respondent indicating one of these diagnoses, regardless of whether they also indicated other pain diagnoses. Respondents were allocated to the pain control group if they reported to have had pain for 3 months or more, but did not indicate the diagnoses CRPS or fibromyalgia. Eight respondents who indicated that they had both CRPS and fibromyalgia were excluded.

#### Demographic and Pain-Related Characteristics

We conducted Kruskal–Wallis tests and Chi-square tests (when more than 20% of cells had expected frequencies below 5, we used Fisher's exact test) to compare the groups regarding demographic and pain-related characteristics.

#### Visual Distortions and Figure Reversals

To evaluate any differences in the number and severity of visual distortions and the number of figure reversals between groups, we performed Kruskal–Wallis tests for each image to compare groups (i.e., CRPS, fibromyalgia, pain control, and pain-free control) regarding the total visual distortions score (for the striped patterns) and the number of figure reversals (for the reversible figures).

Based on previous research, we considered it likely that all participants who completed the task as instructed should perceive at least one reversal for at least one figure (Cohen et al., 2012; Hall et al., 2011). Therefore, for the analysis of the number of figure reversals, we excluded respondents who did not click at least once for at least one figure, because we assumed that those respondents were prevented from clicking due to technical or logistical factors and/or had not understood the instructions. This was the case for two respondents with CRPS, one with fibromyalgia, one with other pain, and two pain-free controls.

#### Pain, Discomfort, and Distress Elicited by Striped Patterns and Reversible Figures

To evaluate any differences in visual discomfort between groups, we performed Kruskal–Wallis tests for each image to compare groups (i.e., CRPS, fibromyalgia, pain control, and pain-free control) regarding their reported change in pain, discomfort, and distress.

#### Light and Pattern Sensitivity in Daily Life

First, we compared scores on the L-VISS and Visual Discomfort Scale between groups (i.e., CRPS, fibromyalgia, pain control, and pain-free control) using Kruskal–Wallis tests.

Next, to evaluate the relationships between light and pattern sensitivity in daily life situations and pain, discomfort, and distress induced by the different images, we conducted Spearman correlations between the total scores on the L-VISS and the Visual Discomfort Scale; and the pain, discomfort, and distress change scores separately for all six images. All respondents were included. Spearman's rho was interpreted as small (>0.10), moderate (>0.30), large (>0.50), or very large (>0.70; Dancey & Reidy, 2004).

## Results

### Demographic and Pain-Related Characteristics

We received 349 responses, of which 60 were excluded because respondents did not finish any part of the study, 8 because they reported as having received a diagnosis of both CRPS and fibromyalgia, and 11 because they did not provide correct answers on one or both control question(s). Because of the potential clinical and theoretical benefit in understanding possible cumulative effects of CRPS and fibromyalgia, we report the data from those participants with both conditions along with exploratory analyses in the Supplemental Material. Of the 270 included responses, 57 were assigned to the CRPS group, 74 to the fibromyalgia group, 50 to the pain control group, and 89 to the pain-free control group (Table 1). The pain-free control group completed the study in less time, was younger than the CRPS and fibromyalgia groups and consisted of more men compared to the other groups. Groups did not differ regarding handedness or dyslexia.

**Table 1.** Demographic and pain-related characteristics, median (interquartile range [IQR]; the first quartile subtracted from the third quartile) and frequency (percentage), split per group.

	CRPS	Fibromyalgia	Pain control	Pain-free control	Statistics
*N*	57	74	50	89	
Duration of study, minutes	22.53 (12.58)^4^	21.18 (12.34)^4^	21.79 (17.07)^4^	17.60 (11.84)	χ^2^(3) = 15.11, *p* = .002
Age	53 (17.00)^4^	51 (18.25)^4^	51 (35.75)	42 (30.50)^1,2^	χ^2^(3) = 17.16, *p* = .001
Sex, % female	52 (91.2%)^4^	67 (90.5%)^4^	39 (78.0%)	62 (69.7%)^1,2^	χ^2^(3) = 16.25, *p* = .001
Handedness^a^					χ^2^(3) = 2.31, *p* = .510
- Left	7 (12.3%)	7 (9.5%)	4 (8.0%)	13 (14.6%)	
- Right	47 (82.5%)	64 (86.5%)	46 (92.0%)	75 (84.3%)	
- Ambidextrous	3 (5.3%)	3 (4.1%)	0	1 (1.1%)	
Dyslexia^a^					χ^2^(3) = 5.07, *p* = .167
- No	48 (84.2%)	62 (83.8%)	36 (76.6%)	65 (73.9%)	
- Maybe/don't know	7 (12.3%)	7 (9.5%)	8 (17.0%)	15 (17.0%)	
- Yes	2 (3.5%)	5 (6.8%)	3 (6.4%)	8 (9.1%)	
Pain duration, in years	6.25 (9.58)^2^	10.46 (16.27)^1^	8.63 (13.75)	-	χ^2^(2) = 8.34, *p* = .015
Hours of pain per day	24 (12)^3^	18 (12)^3^	6 (12)^1,2^	-	χ^2^(2) = 32.30, *p* < .001
Number of pain-related medical diagnoses	2 (2)^2,4^	4 (3)^1,3,4^	2 (2)^2,4^	0 (1)^1,2,3^	χ^2^(3) = 148.74, *p* < .001
Pain, 0–10	7 (3)^2,3,4^	6 (2)^1,3,4^	5 (3)^1,2,4^	0 (1)^1,2,3^	χ^2^(3) = 163.18, *p* < .001
Distress, 0–10	5 (6)^4^	4 (4)^4^	3 (4)^4^	0 (1)^1,2,3^	χ^2^(3) = 97.03, *p* < .001
Discomfort, 0–10	7 (3)^4^	7 (3)^4^	6 (4)^4^	1 (2)^1,2,3^	χ^2^(3) = 150.31, *p* < .001

Abbreviation: CRPS = complex regional pain syndrome.

^a^ As cells had little counts, for handedness we combined the “left” and “ambidextrous” categories, and for dyslexia the “Maybe/don't know” and “Yes” categories for statistical comparisons. Post-hoc tests indicating that the group differs from ^1^CRPS, ^2^fibromyalgia, ^3^pain control, or ^4^pain-free control.

Respondents who reported as having received a diagnosis of fibromyalgia reported the highest number of pain-related medical diagnoses of all groups, and reported to have pain for more years than respondents who reported as having received a diagnosis of CRPS. Respondents who reported as having received a diagnosis of CRPS and pain controls did not differ from each other regarding the number of pain-related diagnoses or the number of years that they reported to be in pain. The specific (comorbid) medical diagnoses are listed in Supplemental Table S2.

Respondents who reported as having received a diagnosis of CRPS or fibromyalgia reported to have more hours of pain per day compared to the pain control group. The baseline level of pain was highest in the CRPS group, followed by the fibromyalgia group, the pain controls, and the pain-free controls. The three pain groups reported similar baseline levels of distress and discomfort, which were higher than in the pain-free control group. The locations of the pain are listed in Supplemental Table S1. For all body parts, more respondents who reported as having received a diagnosis of fibromyalgia reported to experience pain in that body part compared to the CRPS and pain control groups.

### Striped Patterns

Most respondents looked at the images the entire time they were on screen and there was no difference between groups regarding the percentage of respondents who reported to have not looked away from the grey circle (CRPS: 98.2%, fibromyalgia: 100%, pain control: 98.0%, pain-free: 97.8%; χ^2^(3) = 1.59, *p* = .661), the 0.3 cpd stripes (CRPS: 98.2%, fibromyalgia: 100%, pain control: 98.0%, pain-free: 100%; χ^2^(3) = 3.09, *p* = .378), and the 2.3 cpd stripes (CRPS: 98.2%, fibromyalgia: 98.6%, pain control: 98.0%, pain-free: 100%; χ^2^(3) = 1.61, *p* = .656). See Supplemental Table S3 for the reasons that were provided for looking away.

#### Visual Distortions

**Figure 1.**
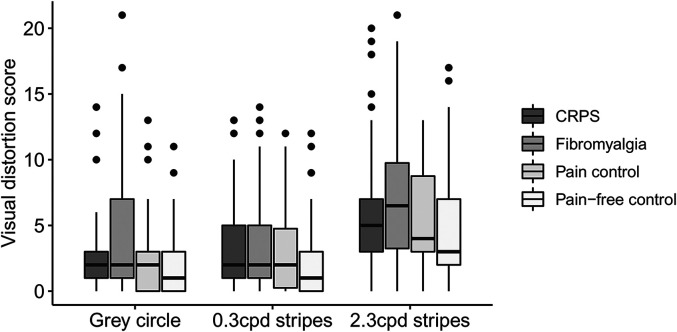
Boxplots depicting the total visual distortion score (ranging from 0 to 21) for the grey circle, the 0.3 cpd striped pattern, and the 2.3 cdp striped pattern, split for respondents with complex regional pain syndrome (CRPS; *N* = 57), fibromyalgia (*N* = 74), pain controls (*N* = 50), and pain-free controls (*N* = 89). The thick line in the middle is the median. The top and bottom box lines show the first and third quartiles. The whiskers show the maximum and minimum values, with the exceptions of outliers (filled black circles). Supplemental Figure 1 includes data from the eight participants who reported as having CRPS and fibromyalgia.

The groups differed regarding the visual distortion score for the grey circle, χ^2^(3) = 11.58, *p* = .009 (Figure 1). Respondents with fibromyalgia reported more visual distortions for the grey circle than pain-free controls (*U* = 2324.5, *r* = −.26). There were no differences in visual distortion score for the grey circle between the other groups (CRPS vs. fibromyalgia: *U* = 1785, *r* = −.13; CRPS vs. pain control: *U* = 1281.5, *r* = −.09; CRPS vs. pain-free: *U* = 2181.5, *r* = −.12; fibromyalgia vs. pain control: *U* = 1408.5, *r* = −.20; pain control vs. pain-free: *U* = 2128, *r* = −.04).

The groups differed regarding the visual distortion score for the 0.3 cpd stripes, χ^2^(3) = 13.53, *p* = .004. Respondents with fibromyalgia (*U* = 2371, *r* = −.24) and respondents with CRPS (*U* = 1791.5, *r* = −.25) reported more visual distortions for the 0.3 cpd striped pattern than pain-free controls. There were no differences in the visual distortion score for the 0.3 cpd striped pattern between the other groups (CRPS vs. fibromyalgia: *U* = 2070.5, *r* = −.02; CRPS vs. pain control: *U* = 1350, *r* = −.05; fibromyalgia vs. pain control: *U* = 1772.5, *r* = −.04; pain control vs. pain-free: *U* = 1728.5, *r* = −.19).

The groups differed regarding the total visual distortion score for the 2.3 cpd stripes, χ^2^(3) = 14.86, *p* = .002. Respondents with fibromyalgia reported more visual distortions for the 2.3 cpd stripes than pain-free controls (*U* = 2223, *r* = −.28). There were no differences in the visual distortion score for the 2.3 cpd stripes between the other groups (CRPS vs. fibromyalgia: *U* = 1829, *r* = −.11; CRPS vs. pain control: *U* = 1315*, r* = −.07; CRPS vs. pain-free: *U* = 1953, *r* = −.19; fibromyalgia vs. pain control: *U* = 1497.5, *r* = −.16; pain control vs. pain-free: *U* = 1819.5, *r* = −.15).

To summarize, for all images, respondents who reported as having received a diagnosis of fibromyalgia reported more visual distortions than pain-free controls. Respondents who reported as having received a diagnosis of CRPS reported more visual distortions than pain-free controls only for the 0.3 cpd stripes. Respondents with fibromyalgia, CRPS, and pain controls did not differ from each other regarding the number of reported visual distortions.

#### Pain, Discomfort, and Distress

**Figure 2.**
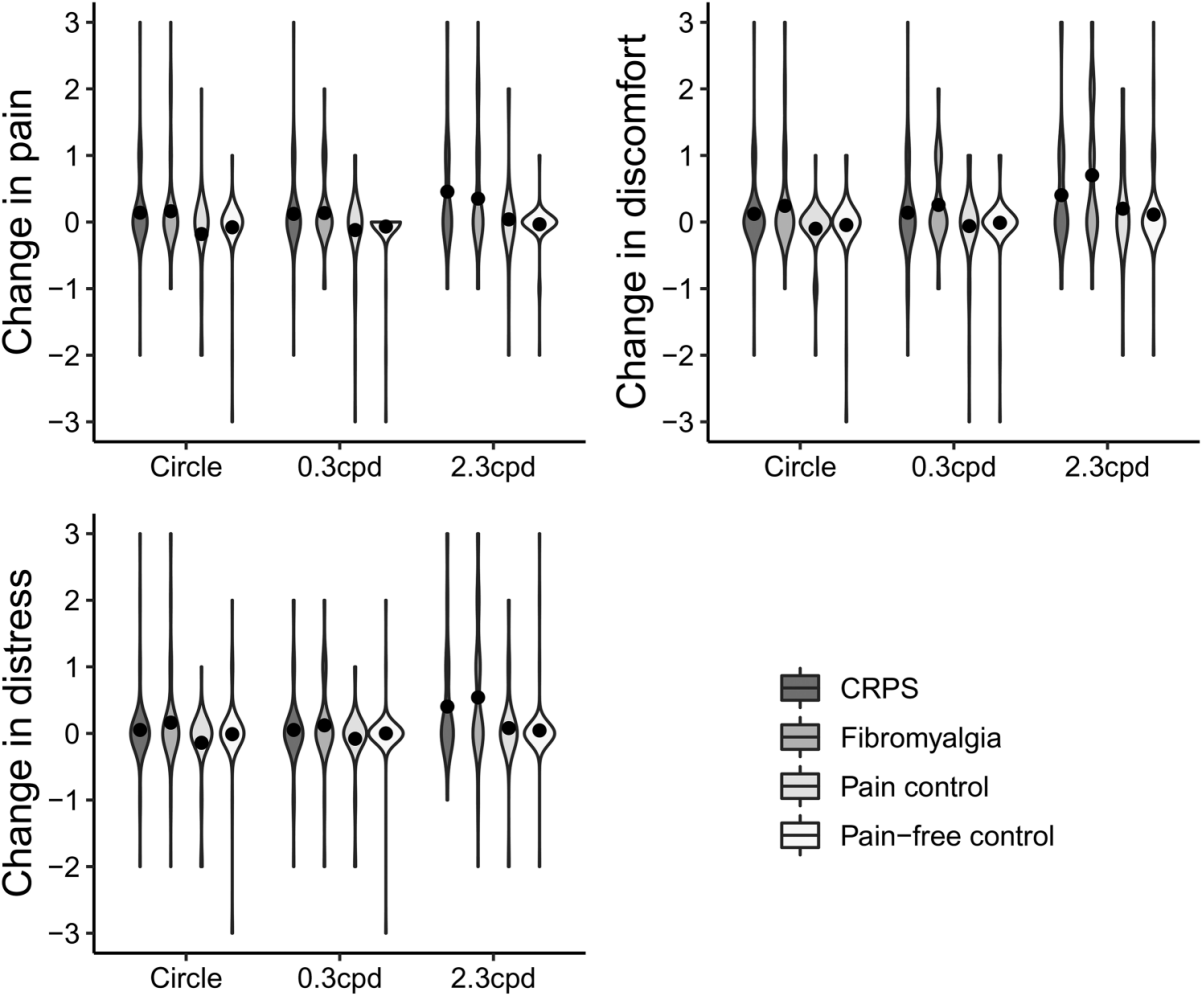
Violin plots and mean scores (black dots) depicting the change in pain, discomfort, and distress for the grey circle, the 0.3 cpd striped pattern, and the 2.3 cdp striped pattern, on a scale from −3 (severe decrease) to +3 (severe increase), split for respondents with complex regional pain syndrome (CRPS; *N* = 57), fibromyalgia (*N* = 74), pain controls (*N* = 50), and pain-free controls (*N* = 89). Supplemental Figure 2 includes data from the eight participants who reported as having CRPS and fibromyalgia.

Figure 2 shows changes in pain, discomfort, and distress for the striped patterns, split per group. Since there was only little variance, violin plots were used to depict scores. In Supplemental Table S4, percentages of respondents indicating either a decrease, no change, or increase are depicted for each group.

The change in pain for the grey circle differed between groups, χ^2^(3) = 14.08, *p* = .003. Respondents with CRPS reported an increase in pain for the grey circle compared to the pain controls (*U* = 1112.5, *r* = −.28) and pain-free controls (*U* = 2,181, *r* = −.21). Respondents with fibromyalgia reported an increase in pain for the grey circle compared to pain controls (*U* = 1,505, *r* = −.24). The other groups did not differ from each other regarding pain increase for the grey circle (fibromyalgia vs. CRPS: *U* = 2046.5, *r* = −.04; fibromyalgia vs. pain-free: *U* = 2,957, *r* = −.15; pain control vs. pain-free: *U* = 2020.5, *r* = −.15). The change in pain for the 0.3 cpd stripes differed between groups, χ^2^(3) = 10.41, *p* = .015. Respondents with CRPS (*U* = 2195.5, *r* = −.21) and respondents with fibromyalgia (*U* = 2840.5, *r* = −.20) reported an increase in pain for the 0.3 cpd stripes compared to pain-free controls. The other groups did not differ from each other regarding pain increase for the 0.3 cpd stripes (fibromyalgia vs. CRPS: *U* = 2,096, *r* = −.01; fibromyalgia vs. pain control: *U* = 1562.5, *r* = −.18; CRPS vs. pain control: *U* = 1,210, *r* = −.19; pain control vs. pain-free: *U* = 2,165, *r* = −.05). The change in pain for the 2.3 cpd stripes differed between groups, χ^2^(3) = 23.88, *p* < .001. Respondents with CRPS reported an increase in pain for the 2.3 cpd stripes compared to the pain controls (*U* = 1053, *r* = −.28) and pain-free controls (*U* = 1752.5, *r* = −.37). Respondents with fibromyalgia reported an increase in pain for the 2.3 cpd stripes compared to the pain-free controls (*U* = 2532.5, *r* = −.29). The other groups did not differ from each other regarding pain increase for the 2.3 cpd stripes (fibromyalgia vs. CRPS: *U* =  1,971, *r* = −.06; fibromyalgia vs. pain control: *U* =  1,499, *r* = −.20; pain control vs. pain-free: *U* =  2,177, *r* = −.03).

The change in discomfort for the grey circle differed between groups, χ^2^(3) = 13.93, *p* = .003. Respondents who reported as having received a diagnosis of fibromyalgia reported an increase in discomfort for the grey circle compared to the pain controls (*U* =  1,455, *r* = −.28). The other groups did not differ regarding change in discomfort for the grey circle (fibromyalgia vs. CRPS: *U* = 2009.5, *r* = −.06; fibromyalgia vs. pain-free: *U* =  2,843, *r* = −.20; CRPS vs. pain control: *U* =  1,181, *r* = −.24; CRPS vs. pain-free: *U* = 2301.5, *r* = −.14; pain control vs. pain-free: *U* = 2037.5, *r* = −.14). The change in discomfort for the 0.3 cpd stripes differed between groups, χ^2^(3) = 11.10, *p* = .011. Respondents who reported as having received a diagnosis of fibromyalgia reported an increase in discomfort for the 0.3 cpd stripes compared to the pain-free controls (*U* =  2,657, *r* = −.25). The other groups did not differ regarding change in discomfort for the 0.3 cpd stripes (fibromyalgia vs. CRPS: *U* =  1,906, *r* = −.10; fibromyalgia vs. pain controls: *U* = 1478.5, *r* = −.22; CRPS vs. pain control: *U* =  1,282, *r* = −.11; CRPS vs. pain-free: *U* =  2,319, *r* = −.12; pain control vs. pain-free: *U* =  2,183, *r* = −.03). The change in discomfort for the 2.3 cpd stripes differed between groups, χ^2^(3) = 19.61, *p* < .001. Respondents who reported as having received a diagnosis of fibromyalgia reported an increase in discomfort for the 2.3 cpd stripes compared to the pain controls (*U* =  1,391, *r* = −.24) and pain-free controls (*U* = 2237.5, *r* = −.34). The other groups did not differ regarding change in discomfort for the 2.3 cpd stripes (fibromyalgia vs. CRPS: *U* = 1763.5, *r* = −.16; CRPS vs. pain control: *U* =  1,297, *r* = −.09; CRPS vs. pain-free: *U* = 2105.5, *r* = −.19; pain control vs. pain-free: *U* =  2,062, *r* = −.08).

The change in distress did not differ between groups for the grey circle, χ^2^(3) = 7.40, *p* = .060, nor for the 0.3 cpd stripes, χ^2^(3) = 6.83, *p* = .078. The change in distress for the 2.3 cpd stripes differed between groups, χ^2^(3) = 25.14, *p* < .001. Respondents who reported as having received a diagnosis of fibromyalgia reported an increase in distress for the 2.3 cpd stripes compared to the pain-free controls (*U* = 2274.5*, r* = −.36) and the pain controls (*U* =  1,376, *r* = −.26). Respondents who reported as having received a diagnosis of CRPS reported an increase in distress for the 2.3 cpd stripes compared to the pain-free controls (*U* = 1981.5, *r* = −.30). The other groups did not differ from each other regarding change in distress for the 2.3 cpd stripes (fibromyalgia vs. CRPS: *U* = 1909.5, *r* = −.09; CRPS vs. pain control: *U* =  1,192, *r* = −.19; pain control vs. pain-free: *U* =  2,101, *r* = −.09).

To summarize, respondents who reported as having received a diagnosis of fibromyalgia reported an increase in pain and discomfort for the grey circle, 0.3 cpd stripes, and 2.3 cpd stripes; and an increase in distress for the 2.3 cpd stripes compared to the pain controls and/or pain-free controls. Respondents who reported as having received a diagnosis of CRPS reported an increase in pain for the grey circle, 0.3 cpd stripes, and 2.3 cpd stripes; and an increase in distress for the 2.3 cpd stripes compared to the pain controls and/or pain-free controls. The other groups did not differ from each other.

### Reversible Figures

Most respondents looked at the figures the entire time they were on screen and there was no difference between groups regarding the percentage of respondents who reported not have looked away from the Duck/Rabbit image (CRPS: 96.5%, fibromyalgia: 95.9%, pain control: 100%, pain-free: 98.9%; χ^2^(3) = 3.21, *p* = .361), the Necker Cube (CRPS: 93.0%, fibromyalgia: 95.9%, pain control: 94.0%, pain-free: 91.0%; χ^2^(3) = 1.63, *p* = .653), and the square (CRPS: 98.2%, fibromyalgia: 100%, pain control: 98.0%, pain-free: 96.6%; χ^2^(3) = 2.54, *p* = .459). See Supplemental Table S3 for the reasons that were provided for looking away.

#### Figure Reversals

**Figure 3.**
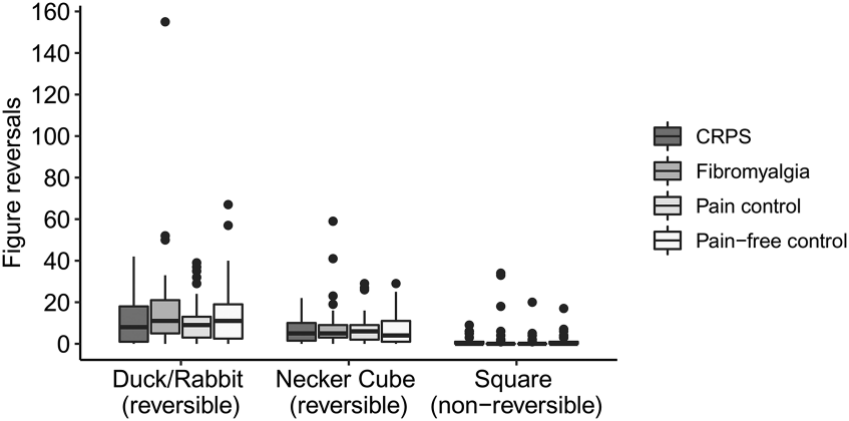
Boxplots depicting the total number of reported figure reversals (measured by mouse clicks) for the reversible Duck/Rabbit figure, the reversible Necker Cube, and the nonreversible square, split for respondents with complex regional pain syndrome (CRPS; *N* = 55), fibromyalgia (*N* = 73), pain controls (*N* = 49), and pain-free controls (*N* = 87). The thick line in the middle is the median. The top and bottom box lines show the first and third quartiles. The whiskers show the maximum and minimum values, with the exceptions of outliers (filled black circles). Supplemental Figure 3 includes data from the eight participants who reported as having CRPS and fibromyalgia.

After excluding respondents who did not click at least once for at least one image, data were available for 55 respondents who reported as having received a diagnosis of CRPS, 73 who reported as having received a diagnosis of fibromyalgia, 49 with other pain, and 87 pain-free controls. Groups did not differ from each other regarding the number of perceived figure reversals for the Duck/Rabbit, χ^2^(3) = 3.07, *p* = .381, the Necker Cube, χ^2^(3) = 1.86, *p* = .603, or the square, χ^2^(3) = 3.41, *p* *=* *.*333 (Figure 3).

#### Pain, Discomfort, and Distress

**Figure 4.**
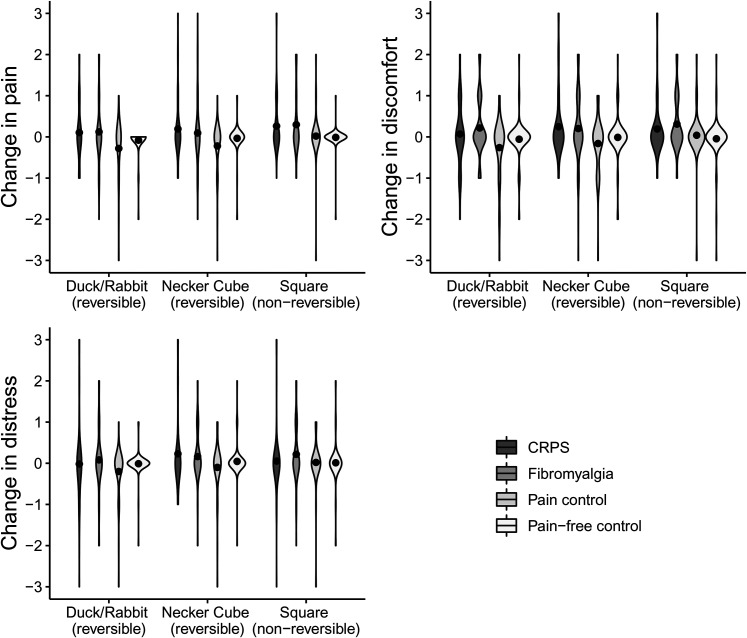
Violin plots and mean scores (black dots) depicting the change in pain, discomfort, and distress for the reversible Duck/Rabbit figure, the reversible Necker Cube, and the nonreversible square, on a scale from −3 (severe decrease) to +3 (severe increase), split for respondents with complex regional pain syndrome (CRPS; *N* = 57), fibromyalgia (*N* = 74), pain controls (*N* = 50), and pain-free controls (*N* = 89). Supplemental Figure 4 includes data from the eight participants who reported as having CRPS and fibromyalgia.

Figure 4 shows changes in pain, discomfort, and distress for the reversible figures, split per group. Since there was only little variance, violin plots were used to depict scores. In Supplemental Table S4, percentages of respondents indicating either a decrease, no change, or increase are depicted for each group.

Groups differed regarding changes in pain for the Duck/Rabbit figure, χ^2^(3) = 9.30, *p* = .026. However, post-hoc testing showed no differences between individual group pairs regarding changes in pain for the Duck/Rabbit figure (fibromyalgia vs. CRPS: *U* =  2,082, *r* = −.01; fibromyalgia vs. pain control: *U* =  1,471, *r* = −.22; fibromyalgia vs. pain-free: *U* =  2,914, *r* = −.15; CRPS vs. pain control: *U* =  1,152, *r* = −.20; CRPS vs. pain-free: *U* = 2281.5, *r* = −.13; pain control vs. pain-free: *U* =  1,987, *r* = −.16). Groups differed regarding changes in pain for the Necker Cube, χ^2^(3) = 10.87, *p* = .012. Respondents in the pain control group reported a larger *decrease* in pain for the Necker Cube relative to respondents who reported as having received a diagnosis of CRPS (*U* =  1,084, *r* = −.28) and pain-free controls (*U* = 1906.5, *r* = −.22). The other groups did not differ from each other regarding changes in pain for the Necker Cube (fibromyalgia vs. CRPS: *U* =  1,991, *r* = −.06; fibromyalgia vs. pain control: *U* =  1,528, *r* = −.19; fibromyalgia vs. pain-free: *U* =  3,120, *r* = −.07; CRPS vs. pain-free: *U* =  2,244, *r* = −.19). Groups differed regarding changes in pain for the square, χ^2^(3) = 16.02, *p* = .001. Respondents who reported as having received a diagnosis of CRPS (*U* =  2,051, *r* = .31) or fibromyalgia (*U* =  2,715, *r* = .26) reported a larger increase in pain for the square compared to the pain-free controls. The other groups did not differ from each other regarding changes in pain for the square (fibromyalgia vs. CRPS: *U* = 2106.5, *r* = 0; fibromyalgia vs. pain control: *U* = 1595.5, *r* = −.17; CRPS vs. pain control: *U* = 1,213, *r* = −.20; pain-controls vs. pain-free: *U* =  2,137, *r* = −.09).

Groups differed regarding changes in discomfort for the Duck/Rabbit figure, χ^2^(3) = 12.73, *p* = .005. Respondents who reported as having received a diagnosis of fibromyalgia reported a larger increase in discomfort for the Duck/Rabbit compared to the pain controls (*U* =  1,348, *r* = −.28) and the pain-free controls (*U* =  2,704, *r* = −.21). The other groups did not differ from each other regarding changes in discomfort for the Duck/Rabbit figure (fibromyalgia vs. CRPS: *U* =  1,932, *r* = −.08; CRPS vs. pain control: *U* = 1160.5, *r* = −.19; CRPS vs. pain-free: *U* = 2316.5, *r* = −.12; pain-controls vs. pain-free: *U* = 1969.5, *r* = −.14). Groups differed regarding changes in discomfort for the Necker Cube, χ^2^(3) = 11.21, *p* = .011. There were, however, no post-hoc differences between groups regarding discomfort changes for the Necker Cube (fibromyalgia vs. CRPS: *U* =  2,044, *r* = −.03; fibromyalgia vs. pain control: *U* =  1,412, *r* = −.23; fibromyalgia vs. pain-free: *U* =  2,811, *r* = −.16; CRPS vs. pain control: *U* = 1112.5, *r* = −.24; CRPS vs. pain-free: *U* =  2,255, *r* = −.14; pain-controls vs. pain-free: *U* =  1,966, *r* = −.14). Groups differed regarding changes in discomfort for the square, χ^2^(3) = 18.12, *p* < .001. Respondents who reported as having received a diagnosis of fibromyalgia reported a larger increase in discomfort for the square compared to the pain-free controls (*U* =  2,466, *r* = −.31). The other groups did not differ from each other regarding changes in discomfort for the square (fibromyalgia vs. CRPS: *U* = 1843.5, *r* = −.14; fibromyalgia vs. pain control: *U* = 1514.5, *r* = −.21; CRPS vs. pain control: *U* = 1356.5, *r* = −.07; CRPS vs. pain-free: *U* = 2226, *r* = −.21; pain-controls vs. pain-free: *U* = 2052.5, *r* = −.13).

Groups did not differ regarding change in distress for the Duck/Rabbit figure, χ^2^(3) = 7.56, *p* = .056, nor for the Necker Cube, χ^2^(3) = 6.39, *p* = .094. Groups differed regarding change in distress for the square, χ^2^(3) = 8.29, *p* = .040, but none of the post-hoc comparisons were significant (fibromyalgia vs. CRPS: *U* = 1786.5, *r* = −.18; fibromyalgia vs. pain control: *U* = 1629.5, *r* = −.13; fibromyalgia vs. pain-free: *U* = 2,780, *r* = −.19; CRPS vs. pain control: *U* =  1,354, *r* = −.07; CRPS vs. pain-free: *U* = 2516.5, *r* = −.01; pain-controls vs. pain-free: *U* = 2127.5, *r* = −.07).

To summarize, respondents who reported as having received a diagnosis of CRPS or fibromyalgia reported a larger increase in pain for the square compared to the pain-free controls. Respondents with fibromyalgia reported a larger increase in discomfort for the square and Duck/Rabbit figure than the pain controls and/or the pain-free controls. The Duck/Rabbit figure and Necker Cube did not increase pain or distress in respondents with CRPS or fibromyalgia. However, respondents in the pain control group reported a larger *decrease* in pain for the Necker Cube relative to respondents who reported as having received a diagnosis of CRPS and pain-free controls.

### Light and Pattern Sensitivity in Daily Life

**Figure 5.**
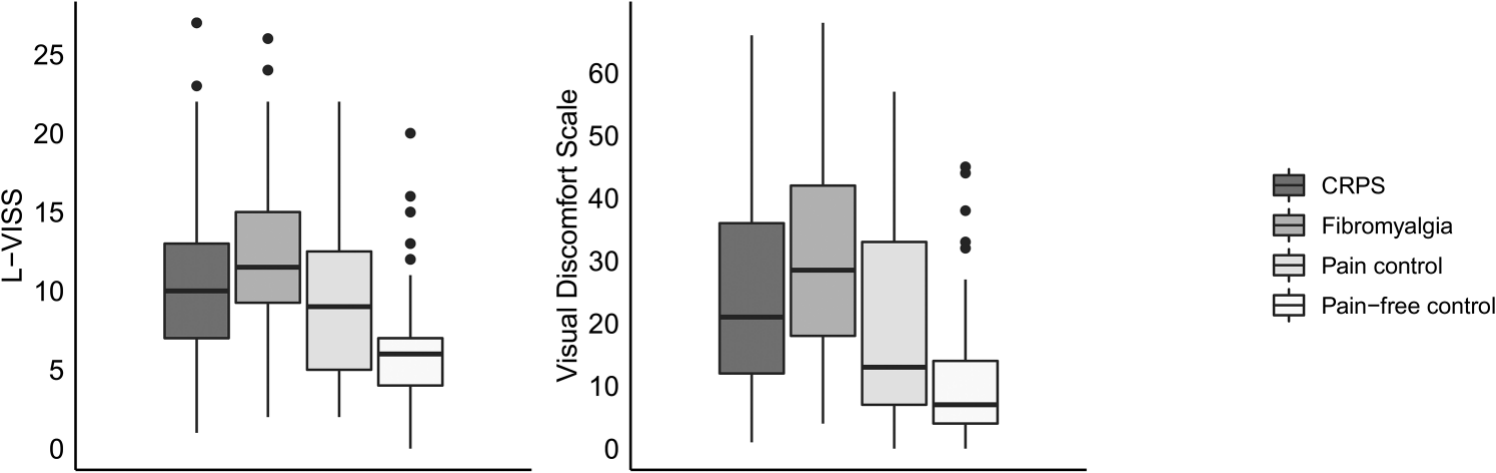
Boxplots depicting the Leiden Visual Sensitivity Scale (L-VISS) and Visual Discomfort Scale, split for respondents with complex regional pain syndrome (CRPS; *N* = 57), fibromyalgia (*N* = 74), pain controls (*N* = 50), and pain-free controls (*N* = 89). The thick line in the middle is the median. The top and bottom box lines show the first and third quartiles. The whiskers show the maximum and minimum values, with the exceptions of outliers (filled black circles). Supplemental Figure 5 includes data from the eight participants who reported as having CRPS and fibromyalgia.

Figure 5 depicts the L-VISS and Visual Discomfort Scale scores per group. The groups differed regarding light and pattern sensitivity in daily life as measured with the L-VISS, χ^2^(3) = 70.23, *p* < .001, and the Visual Discomfort Scale, χ^2^(3) = 80.98, *p* < .001. Respondents who reported as having received a diagnosis of fibromyalgia obtained higher scores than respondents who reported as having received a diagnosis of CRPS (L-VISS: *U* = 1625.5, *r* = .16, Visual Discomfort Scale: *U* = 1591.5, *r* = .21), pain controls (L-VISS: *U* = 1085.5, *r* = −.35, Visual Discomfort Scale: *U* = 1000.5, *r* = −.35), and pain-free controls (L-VISS: *U* = 896.5, *r* = −.62, Visual Discomfort Scale: *U* = 700.5, *r* = −.68). There was no difference between respondents who reported as having received a diagnosis of CRPS and pain controls. Respondents who reported as having received a diagnosis of CRPS obtained higher scores than the pain-free controls (L-VISS: *U* =  1,226*, r* = −.44, Visual Discomfort Scale: *U* =  1,057, *r* = −.49). Pain controls obtained higher scores than pain-free controls (L-VISS: *U* = 1458.5, *r* = −.29, Visual Discomfort Scale: *U* =  1,334, *r* = −.29).

For the grey circle, there were small positive relationships between the L-VISS and changes in pain, discomfort, and distress (Table 2). No such relationships were present for the Visual Discomfort Scale. Both the L-VISS and Visual Discomfort Scale showed small positive relationships with changes in pain, discomfort, and distress for the 0.3 cpd striped pattern, and small to moderate positive relationships for the 2.3 striped pattern. The pain, discomfort, and distress change scores for the reversible Duck-Rabbit figure were not related to L-VISS and Visual Discomfort Scale scores. The pain, discomfort, and distress change scores for the Necker Cube and Square showed small positive relationships with the L-VISS and Visual Discomfort Scale.

**Table 2.** Spearman correlation coefficients between the change in pain, discomfort, and distress ratings for the different images and visual sensitivity in daily life situations, as measured with the Leiden Visual Sensitivity Scale (L-VISS) and Visual Discomfort Scale. All respondents were included (*N* = 270).

		L-VISS	Visual discomfort scale
Striped patterns			
Grey circle	Pain	0.20*	0.12
	Discomfort	0.17*	0.12
	Distress	0.14*	0.12
0.3 cpd stripes	Pain	0.18*	0.15*
	Discomfort	0.24*	0.18*
	Distress	0.19*	0.15*
2.3 cpd stripes	Pain	0.25**	0.24**
	Discomfort	0.32**	0.29**
	Distress	0.34**	0.29**
Reversible figures			
Duck/Rabbit	Pain	0.08	−0.03
	Discomfort	0.07	−0.01
	Distress	0	−0.05
Necker Cube	Pain	0.15*	0.10
	Discomfort	0.20**	0.14*
	Distress	0.22**	0.15*
Square	Pain	0.16*	0.17*
	Discomfort	0.20**	0.23**
	Distress	0.14*	0.15*

*p < .05; **p < .001.

## Discussion

The aim of this study was to investigate visual sensitivity in people who reported as having received a diagnosis of CRPS or fibromyalgia compared to people with other chronic pain conditions or no chronic pain. With regard to the striped patterns, we found that people who reported as having received a diagnosis of CRPS reported more visual distortions while viewing a 0.3 cpd striped pattern and an increase in pain and distress while viewing a 2.3 cpd striped pattern and a grey circle compared to pain-related controls and pain-free controls. People who reported as having received a diagnosis of fibromyalgia reported more visual distortions, and an increase in pain, discomfort, and/or distress while viewing 0.3 and 2.3 cpd striped patterns, and a grey circle compared to pain-related controls and pain-free controls. The pain-related controls did not report more visual distortions or visual discomfort compared to the pain-free controls for any of the images.

In people with (only) migraine, visual discomfort is especially pronounced for striped patterns with a high (e.g., 2.3 cpd) versus low (e.g., 0.3 cpd) spatial frequency (Conlon et al., 2001; Wilkins et al., 1984). In the current study, people who reported as having received a diagnosis of CRPS (of whom 14% reported to have migraine) or fibromyalgia (of whom 40.5% reported to have migraine) experienced visual discomfort for the low spatial frequency pattern and grey circle as well as the high spatial frequency pattern. This suggests that some of the group differences may be caused by differences in pain anticipation in the CRPS and fibromyalgia groups. According to the generalized hypervigilance hypothesis in fibromyalgia, heightened sensitivity to pain can be the consequence of increased attention to external stimulation and a preoccupation with pain sensations. The hypervigilance to painful stimuli extends to nonnoxious stimuli, such as visual stimuli (McDermid et al., 1996; Rollman, 2009; Rollman & Lautenbacher, 1993). The information about the study at the start and the type of questions being asked, might have encouraged such hypervigilance by suggesting that pain, discomfort, and distress were expected to change. However, against this interpretation, viewing the reversible Duck/Rabbit figure did not increase pain in people who reported as having received a diagnosis of CRPS or fibromyalgia, but only increased discomfort in people with fibromyalgia compared to the pain-related controls and pain-free controls. This is contrary to our expectations and the results of Cohen et al. (2012), who showed that about half of people with CRPS reported increased pain while viewing the reversible Duck/Rabbit. We also found a group difference for the reversible Necker Cube, which is potentially driven by a *decrease* in pain for the pain controls relative to an *increase* or no change in pain for people who reported as having received a diagnosis of CRPS and pain-free controls. The same pattern was seen by Hall et al. (2011). This decrease in pain could relate to distraction relieving the pain in the pain-related controls, but not for those with CRPS or fibromyalgia. Distraction was spontaneously mentioned by some people in the free text box (Supplemental Table S5), although not to different extents across groups. Previous researchers who used similar reversible figures suggested that the level of unpleasantness experienced by people with CRPS could potentially be explained by a high figure reversal rate (Cohen et al., 2012; Hall et al., 2011). However, people who reported as having received a diagnosis of CRPS or fibromyalgia did not report more figure reversals while viewing the reversible Duck/Rabbit or Necker Cube compared to pain-related controls and pain-free controls. Finally, the nonreversible square induced a larger increase in pain and/or discomfort in people who reported as having received a diagnosis of CRPS or fibromyalgia compared to pain-free controls. An increase in pain while viewing the nonreversible square has also been observed in several participants with CRPS by previous studies (Cohen et al., 2012; Hall et al., 2011), and might be related to sensitivity to geometric shapes (Wilkins et al., 2007, 2018).

People in the pain groups reported a higher degree of light and pattern sensitivity in daily life compared to pain-free controls, which was highest for people who reported as having received a diagnosis of CRPS or fibromyalgia. This could not be explained by the presence of dyslexia. Light and pattern sensitivity in daily life showed small to moderate positive relationships with pain, discomfort, and distress for the 2.3 cpd striped pattern, and small positive relationships with pain, discomfort, and distress for the grey circle, 0.3 cpd striped pattern, reversible Necker Cube, and nonreversible square. Light and pattern sensitivity in daily life was not related to pain, discomfort, or distress for the reversible Duck/Rabbit.

Our exploratory analysis of people reporting both fibromyalgia and CRPS indicated that self-reported visual discomfort was higher than for people with only one of these conditions, suggesting that there might be additive effects of these conditions. However, this observation should be treated with caution, due to the very low sample size and the fact that this group did not show such additive effects for the experimental measures of visual discomfort.

Altogether, these results suggest that people who reported as having received a diagnosis of CRPS or fibromyalgia have higher somatic sensitivity than pain-related controls and pain-free controls for striped patterns and geometric shapes, regardless of their spatial frequency. This group difference is not seen for more natural, complex images involving higher-order visual processing (i.e., the reversible Duck/Rabbit). Furthermore, visual discomfort for striped patterns and geometric shapes, especially those with a high spatial frequency, is related to light and pattern sensitivity in daily life. This is in line with findings of people with migraine, who report greater discomfort during the Pattern Glare Test and higher light and pattern sensitivity in daily life as measured with the L-VISS and Visual Discomfort Scale compared to pain-free controls (Perenboom et al., 2018; Shepherd et al., 2013). Our results fit with the hypothesis that people with CRPS or fibromyalgia have a hyperactive or over sensitive central nervous system (central sensitization) (Adams & Turk, 2015; Ji & Woolf, 2001; Yunus, 2008); and that geometric shapes and striped patterns lead to enhanced cortical neural activity in the early visual systems compared to more natural images, such as the Duck/Rabbit figure. Aspects of spatial coding in the visual system might be matched to the natural environment, and the visual system might not be well equipped to process stimuli that are very different from natural scenes (Hibbard & O’Hare, 2015; Juricevic et al., 2010; Monger et al., 2015; O’Hare & Hibbard, 2011).

### Limitations

To maximize sample size and in order to include people who live distant from our lab and/or are not able to travel, we conducted an online survey which we distributed internationally. One limitation is that some of the English terms might not be consistent across countries or fully understood by nonnative speakers. Second, we had less control over the testing environment than is possible for in-person laboratory studies (e.g., the type of computer used, viewing distance, distractions, and whether respondents understood the instructions). To partially overcome these issues, we blocked participation through mobile devices, only included images for which the effects were not dependent on viewing distance, asked participants whether they had viewed the image the entire time it was on screen, and included control questions to measure participant engagement. Furthermore, we compared conditions within participants, thus variability in, for example, screens would not have had an impact on our results. For the group comparisons, we expect a similar amount of variability in screens between groups and, therefore, we do not expect this to account for our findings. We had no control on how long respondents spent on the study and we found that the three pain groups needed more time than the pain-free control group to complete the study (at least partly because they completed additional questions about their pain). Possibly, looking at a screen for longer or longer time-on-task in general could lead to more fatigue, discomfort, distress, and/or pain. The difference in completion time was in the range of minutes and we do not expect our results to be fully explained by it. In addition, the CRPS and fibromyalgia groups needed a similar amount of time for the study as the pain control group. Therefore, differences between these groups cannot be explained by longer time-on-task. Third, groupings were based on self-reported diagnoses rather than independent clinical evaluation. However, in previous research using a similar recruitment strategy, most participants reported having received their diagnoses from an appropriately qualified clinician (Ten Brink et al., 2020). Furthermore, the patterns of demographic and pain-related characteristics in our study are consistent with those found in previous research. For example, there was a higher proportion of females in the pain groups than the pain-free control group (Borchers & Gershwin, 2015; Marinus & van Hilten, 2006; Mills et al., 2019; Van Hecke et al., 2013), respondents who reported as having received a diagnosis of CRPS most frequently reported their limb(s) as being most painful as opposed to other body parts, whereas respondents who reported as having received a diagnosis of fibromyalgia reported widespread pain (Wurtman, 2010). Nevertheless, it is possible that some respondents who reported as having received a diagnosis of CRPS did not fulfil the CRPS criteria at the time, whereas some respondents in the pain control group did fulfil the CRPS criteria, but never had received any diagnosis.

Groups were not comparable regarding demographic characteristics. The pain-free control group was younger than the pain groups, and there were fewer females in this group. However, previous research found no difference between males and females regarding pattern-related visual discomfort; and either no effect of age or *less* pattern glare in older versus younger participants (Bargary, Jia, et al., 2015; Evans & Stevenson, 2008; Ten Brink et al., 2021). Thus, the lower levels of visual discomfort in the pain-free control group compared to the pain groups can most likely not be explained by differences in sex and age. Alternative factors that might have accounted for differences between the pain groups could be pain chronicity, pain intensity, medication, uncorrected or inappropriately corrected vision, other ocular conditions and diseases, or sleep deprivation. About 67%–88% of people with chronic pain report that their sleep is disrupted (Finan et al., 2013), and a night of sleep loss enhances visual discomfort (Dyakova et al., 2019). Furthermore, fibromyalgia and other chronic pain disorders are associated with visual disturbances (blurred vision) and other ocular conditions that may cause visual distress (Aykut et al., 2018; Schuster et al., 2018; Ten Brink et al., 2020; Zdebik et al., 2021). Further research could aim to better evaluate whether the visual disturbances that we found here are district from or secondary to such conditions.

Another limitation is that migraine could have been a factor in the differences between groups. However, when we repeated analyses without people who reported a diagnosis of migraine, results were comparable. In addition, viewing a bright computer screen in itself could have caused visual discomfort in people with fibromyalgia and CRPS. Although this may have increased the visual discomfort, this is not likely the only explanation, as groups did not differ regarding visual discomfort while viewing the Duck/Rabbit figure.

Respondents were asked to fixate the center of the image only for the part with the striped patterns and not for the part with the reversible images. In both parts, eye movements were not measured and we did not compare eye movement patterns between groups. Eye movement patterns play a role in perceived perceptual alternations, visual distortions, and visual discomfort. For example, there is a relation between eye position on the Necker cube and reported perceptual alternations (e.g., Einhauser et al., 2004; Polgári et al., 2020). Furthermore, microsaccades have been related to reported illusions of motion for repetitive patterns, although there is evidence for an additional cortical component of the perceived illusion (e.g., Kumar & Glaser, 2006; O’Hare, 2017; Troncoso et al., 2008). Possibly, respondents who reported as having received a diagnosis of fibromyalgia, and to a lesser extent respondents with CRPS, executed different eye movement patterns than the other groups which could underly differences in reported discomfort, distress, and/or pain. Although some studies have reported oculomotor disturbances in people with fibromyalgia, such as reduced saccade velocity and altered smooth pursuit (Rosenhall et al., 1987, 1996), it is unclear whether oculomotor abnormalities have played a role in visual distortions and visual discomfort in the current study.

Finally, to assess effects of low versus high spatial frequency on visual distortions and visual discomfort, we only used two different square-wave gratings (i.e., 0.3 and 2.3 cpd). To gain more insight into effects of spatial frequency, more stimuli with a wider range of spatial frequencies should be used in future studies, preferably in a setting where viewing distance can be controlled for.

### Conclusion

People who reported as having received a diagnosis of CRPS or fibromyalgia experienced higher levels of visual sensitivity compared to people with other pain conditions or no chronic pain while viewing striped patterns or geometric shapes, with no clear effect of spatial frequency. Understanding which image features induce negative sensations is beneficial in a clinical context where sensory difficulties could be accommodated (e.g., avoiding repetitive geometric patterns in pain management clinics). Future studies could evaluate whether precision spectral filters that are thought to suppress visual cortical activity can offer relief in people with CRPS and fibromyalgia similarly as in other central nervous system disorders such as photosensitive epilepsy and migraine (Monger et al., 2015; Shepherd et al., 2013; Wilkins et al., 2007).

## Supplemental Material

sj-pdf-1-pec-10.1177_03010066211072641 - Supplemental material for Visual Sensitivity in Complex Regional Pain Syndrome and Fibromyalgia: An Online Study Click here for additional data file. Supplemental material, sj-pdf-1-pec-10.1177_03010066211072641 for Visual Sensitivity in Complex Regional Pain Syndrome and Fibromyalgia: An Online Study by Antonia F. Ten Brink and Janet H. Bultitude in Perception
