# Supplementary material for: Visual Sensitivity in Complex Regional Pain Syndrome and Fibromyalgia: An Online Study
Source: Perception. 2022 Mar 3;51(3):187–209. doi: 10.1177/03010066211072641 (PMC8958570; doi:10.1177/03010066211072641)
Supplement: sj-pdf-1-pec-10.1177_03010066211072641 - Supplemental material for Visual Sensitivity in Complex Regional Pain Syndrome and Fibromyalgia: An Online Study [file sj-pdf-1-pec-10.1177_03010066211072641.pdf]

Table S1. Numbers and percentages of painful locations split by group. Note that respondents could report multiple diagnoses, thus percentages do not sum to 100.

	**CRPS (*N* = 57)**	**Fibromyalgia (*N* = 74)**	**Pain controls (*N* = 50)**	**Statistics**
Arm/hand (including shoulder, elbow, wrist, fingers)	37 (64.9%)[2]	71 (95.9%)[1,3]	30 (60.0%)[2]	$\chi^2(2) = 27.18$, $p < .001$
Leg/foot (including hip, knee, ankle, toes, foot sole)	50 (87.7%)	73 (98.6%)[3]	41 (82.0%)[2]	$\chi^2(2) = 10.53$, $p = .005$
Back	29 (50.9%)[2]	69 (93.2%)[1,3]	33 (66.0%)[2]	$\chi^2(2) = 30.31$, $p < .001$
Head	20 (35.1%)[2]	58 (78.4%)[1,3]	14 (28.0%)[2]	$\chi^2(2) = 38.55$, $p < .001$
Face (including ears, eyes, jaw, teeth)	18 (31.6%)[2]	52 (70.3%)[1,3]	14 (28.0%)[2]	$\chi^2(2) = 28.79$, $p < .001$
Neck	28 (49.1%)[2]	58 (78.4%)[1,3]	18 (36.0%)[2]	$\chi^2(2) = 24.29$, $p < .001$
Chest/ribs	17 (29.8%)[2]	52 (70.3%)[1,3]	11 (22.0%)[2]	$\chi^2(2) = 35.16$, $p < .001$
Stomach/abdomen	13 (22.8%)[2]	52 (70.3%)[1,3]	13 (26.0%)[2]	$\chi^2(2) = 37.81$, $p < .001$
Groin/genitals	10 (17.5%)[2]	31 (41.9%)[1,3]	8 (16.0%)[2]	$\chi^2(2) = 13.95$, $p < .001$
Whole body	7 (12.3%)[2]	49 (66.2%)[1,3]	10 (20.0%)[2]	$\chi^2(2) = 48.51$, $p < .001$
Other	3 (5.3%)	7 (9.5%)	5 (10.0%)	$p = .621$

Abbreviation: Complex Regional Pain Syndrome, CRPS.

Group mean differed significantly from [1]CRPS, [2]fibromyalgia, [3]pain controls, and [4]pain-free controls

Table S2. Numbers and percentages of medical diagnoses split by group. Note that respondents could report multiple diagnoses, thus percentages do not sum to 100.

	**CRPS (*N* = 57)**	**Fibromyalgia (*N* = 74)**	**Pain controls (*N* = 50)**	**Pain-free controls (*N* = 89)**	**Statistics**
CRPS	57 (100%)	0	0	0	-
Fibromyalgia	0	74 (100%)	0	1 (1.1%)	-
Rheumatoid Arthritis	1 (1.8%)[3]	3 (4.1%)	9 (18.0%)[1,4]	0[3]	$p < .001$
Osteoarthritis	10 (17.5%)	17 (23.0%)[4]	13 (26.0%)[4]	4 (4.5%)[2,3]	$p < .001$
Plantar fasciitis	3 (5.3%)	10 (13.5%)	1 (2.0%)	5 (5.6%)	$p = .093$
Hypermobility	5 (8.8%)	9 (12.2%)	8 (16.0%)	3 (3.4%)	$p = .048$
Back pain	14 (24.6%)[4]	31 (41.9%)[4]	23 (46.0%)[4]	7 (7.9%)[1,2,3]	$p < .001$
Migraine	8 (14.0%)[2]	30 (40.5%)[1,4]	11 (22.0%)	9 (10.1%)[1]	$p < .001$
Cluster Headache	1 (1.8%)	4 (5.4%)	3 (6.0%)	3 (3.4%)	$p = .665$
Chronic Fatigue Syndrome	1 (1.8%)[2]	17 (23.0%)[1,3,4]	2 (4.0%)[2]	0[2]	$p < .001$
Neuralgia	3 (5.3%)	6 (8.1%)[4]	3 (6.0%)	0[1]	$p = .025$
Osteoporosis	2 (3.5%)	4 (5.4%)	2 (4.0%)	1 (1.1%)	$p = .450$
Endometriosis	2 (3.5%)	7 (9.5%)	1 (2.0%)	1 (1.1%)	$p = .059$
Irritable bowel syndrome/inflammatory bowel disease	4 (7.0%)[2]	39 (52.7%)[1,3,4]	8 (16.0%)[2]	4 (4.5%)[2]	$p < .001$
Degenerative Disc Disease	2 (3.5%)[3]	10 (13.5%)[4]	10 (20.0%)[1,4]	0[2,3]	$p < .001$
Other (one or more other pain-related diagnosis)	8 (14.0%)[3]	14 (18.9%)[3]	24 (48.0%)[1,2]	-	$\chi^2(2) = 19.00$, $p < .001$
None	0	0	4 (8.0%)	65 (73.0%)	-

Abbreviation: Complex Regional Pain Syndrome, CRPS.

Group mean differed significantly from [1]CRPS, [2]fibromyalgia, [3]pain controls, and [4]pain-free controls.

Table S3. Reasons for people to look away from the image, the percentage of viewing time in brackets, and the reasons that were provided for the "other" category in the free-text box, split per group.

		CRPS	Fibromyalgia	Pain controls	Pain-free controls	Other reasons
**Grey circle**	Pain/discomfort/distress	0	0	0	0	CRPS: looking for further instructions
	Distracted	0	0	1 (70%)	0	Pain-free: watery eyes
	Bored	0	0	0	1 (80%)	
	Other	1 (92%)	0	0	1 (80%)	
**0.3cpd**	Pain/discomfort/distress	1 (31%)	0	0	0	
	Distracted	0	0	1 (62%)	0	
	Bored	0	0	0	0	
	Other	0	0	0	0	
**2.3cpd**	Pain/discomfort/distress	1 (26%)	1 (28%)	0	0	
	Distracted	0	0	1 (missing)	0	
	Bored	0	0	0	0	
	Other	0	0	0	0	
**Duck/Rabbit**	Pain/discomfort/distress	1 (98%)	0	0	0	Fibromyalgia: could see both at the same time, so no change
	Distracted	0	0	0	0	
	Bored	0	2 (38%, 61%)	0	1 (60%)	CRPS: I though the screen had frozen
	Other	1 (80%)	1 (90%)	0	0	
**Necker Cube**	Pain/discomfort/distress	1 (55%)	0	0	0	Pain-free: bored and made me feel dizzy
	Distracted	0	0	0	0	
	Bored	0	0	1 (60%)	2 (19%, 39%)	
	Other	0	0	0	1 (92%)	
**Square**	Pain/discomfort/distress	1 (49%)	1 (20%)	0	0	CRPS: postal delivery; checking if no scripts were blocking the survey; eyes went fuzzy
	Distracted	0	0	1 (80%)	1 (84%)	
	Bored	0	1 (85%)	2 (61%, 64%)	5 (29%, 30%, 41%, 50%, 72%,)	Fibromyalgia: thought nothing would change
	Other	3 (51%, 87%, 88%)	1 (70%)	0	2 (19%, 78%)	Pain-free: bored and made me feel dizzy

Table S4. Percentage of respondents indicating a decrease (severe, moderate, or mild) / no change / increase (mild, moderate, or severe) of pain, discomfort, and distress per image, split per group.

		**CRPS (N = 57)**	**Fibromyalgia (N = 74)**	**Pain controls (N = 50)**	**Pain-free controls (N = 89)**
**Grey circle**	Pain	3.5 / 80.7 / 15.8	5.4 / 81.1 / 13.5	14.0 / 84.0 / 2.0	4.5 / 93.3 / 2.2
	Discomfort	3.5 / 82.5 / 14.0	2.7 / 79.7 / 17.6	10.0 / 88.0 / 2.0	3.4 / 92.1 / 4.5
	Distress	5.3 / 87.7 / 7.0	4.l / 82.4 / 13.5	10.0 / 88.0 / 2.0	3.4 / 91.0 / 5.6
**0.3cpd**	Pain	5.3 / 78.9 / 15.8	6.8 / 75.7 / 17.6	12.0 / 82.0 / 6.0	3.4 / 96.6 / 0
	Discomfort	8.8 / 71.9 / 19.3	5.4 / 67.6 / 27.0	10.0 / 80.0 / 10.0	2.2 / 93.3 / 4.5
	Distress	7.0 / 82.5 / 10.5	5.4 / 77.0 / 17.6	8.0 / 88.0 / 4.0	1.1 / 96.6 / 2.2
**2.3cpd**	Pain	5.3 / 59.6 / 35.1	6.8 / 64.9 / 28.4	8.0 / 84.0 / 8.0	4.5 / 93.3 / 2.2
	Discomfort	5.3 / 63.2 / 31.6	4.1 / 51.4 / 44.6	8.0 / 68.0 / 24.0	3.4 / 84.3 / 12.4
	Distress	3.5 / 68.4 / 28.1	5.4 / 55.4 / 39.2	4.0 / 84.0 / 12.0	1.1 / 95.5 / 3.4
**Duck/Rabbit**	Pain	15.8 / 63.2 / 21.1	12.2 / 68.9 / 18.9	20.0 / 76.0 / 4.0	5.6 / 94.4 / 0
	Discomfort	17.5 / 59.6 / 22.8	10.8 / 63.5 / 25.7	22.0 / 72.0 / 6.0	7.9 / 88.8 / 3.4
	Distress	17.5 / 71.9 / 10.5	10.8 / 71.6 / 17.6	16.0 / 82.0 / 2.0	2.2 / 95.5 / 2.2
**Necker Cube**	Pain	7.0 / 75.4 / 17.5	13.5 / 68.9 / 17.6	18.0 / 80.0 / 2.0	3.4 / 94.4 / 2.2
	Discomfort	5.3 / 77.2 / 17.5	12.2 / 59.5 / 28.4	22.0 / 68.0 / 10.0	6.7 / 85.4 / 7.9
	Distress	7.0 / 78.9 / 14.0	6.8 / 71.6 / 21.6	12.0 / 80.0 / 8.0	1.1 / 93.3 / 5.6
**Square**	Pain	1.8 / 77.2 / 21.1	4.1 / 74.3 / 21.6	2.0 / 92.0 / 6.0	1.1 / 97.8 / 1.1
	Discomfort	3.5 / 82.5 / 14.0	4.1 / 67.6 / 28.4	2.0 / 90.0 / 8.0	5.6 / 91.0 / 3.4
	Distress	7.0 / 86.0 / 7.0	6.8 / 68.9 / 24.3	4.0 / 86.0 / 10.0	4.5 / 89.9 / 5.6

Abbreviation: Complex Regional Pain Syndrome, CRPS.

Table S5. Number of respondents per group who (spontaneously) reported that viewing the image and/or performing the task was distracting them from their pain, discomfort, or distress.

	**CRPS**	**Fibromyalgia**	**Pain controls**	**Pain-free controls**
Striped patterns				
Grey circle	0	1	1	0
0.3cpd pattern	0	0	1	0
2.3cpd pattern	0	1	0	0
Reversible figures				
Duck/Rabbit	1	1	1	2
Necker Cube	0	0	1	1
Square	0	0	0	1

Abbreviation: Complex Regional Pain Syndrome, CRPS.

S1. Additional results on the respondents who reported to have received a diagnosis of CRPS and fibromyalgia

Of all respondents, eight reported to have received a diagnosis of CRPS and fibromyalgia (“CRPS+fibromyalgia”). They were all women, 6 were right-handed and they had a median age of 52 years (IQR = 18.25). None of them reported having dyslexia, three reported “maybe/I don’t know”. The median duration of the time needed to complete the study was 20.63 minutes (IQR = 14.08). The median pain duration of the CRPS+fibromyalgia group was 16.67 years (IQR = 9.25) and the median hours of pain per day was 18 (IQR = 8). The median total number of pain-related diagnoses was 5.50 (IQR = 3). The median level of pain was 7.00 (IQR = 1), the level of distress was 5.50 (IQR = 5), and the level of discomfort was 7.00 (IQR = 2).

All eight respondents with CRPS and fibromyalgia reported to have looked at the striped patterns and bistable images the entire time they were on screen. One respondent with CRPS and fibromyalgia reported to have looked away from the square because it caused to much pain, discomfort, or distress. One respondent with CRPS and fibromyalgia did not click at least on one of the bistable images and was excluded from the analysis on the number of figure reversals.

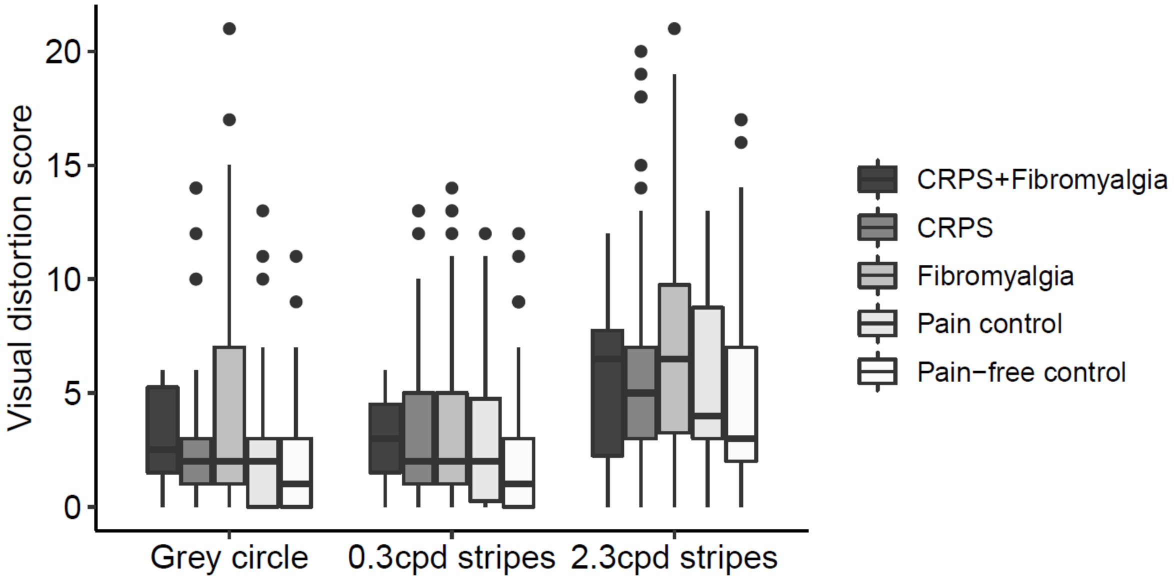

Supplementary Figure 1. Boxplots depicting the total visual distortion score (ranging from 0 to 21) for the grey circle, the 0.3cpd striped pattern, and the 2.3cdp striped pattern, split for respondents with Complex Regional Pain Syndrome (CRPS) and fibromyalgia (N = 8), CRPS (N = 57), fibromyalgia (N = 74), pain controls (N = 50), and pain-free controls (N = 89). The thick line in the middle is the median. The top and bottom box lines show the first and third quartiles. The whiskers show the maximum and minimum values, with the exceptions of outliers (circles). Exploratory Mann Whitney Tests (i.e. not corrected for multiple comparisons) showed that the CRPS+fibromyalgia group did not significantly differ from any of the other groups regarding reported visual distortions (*ps* ≥ .213).

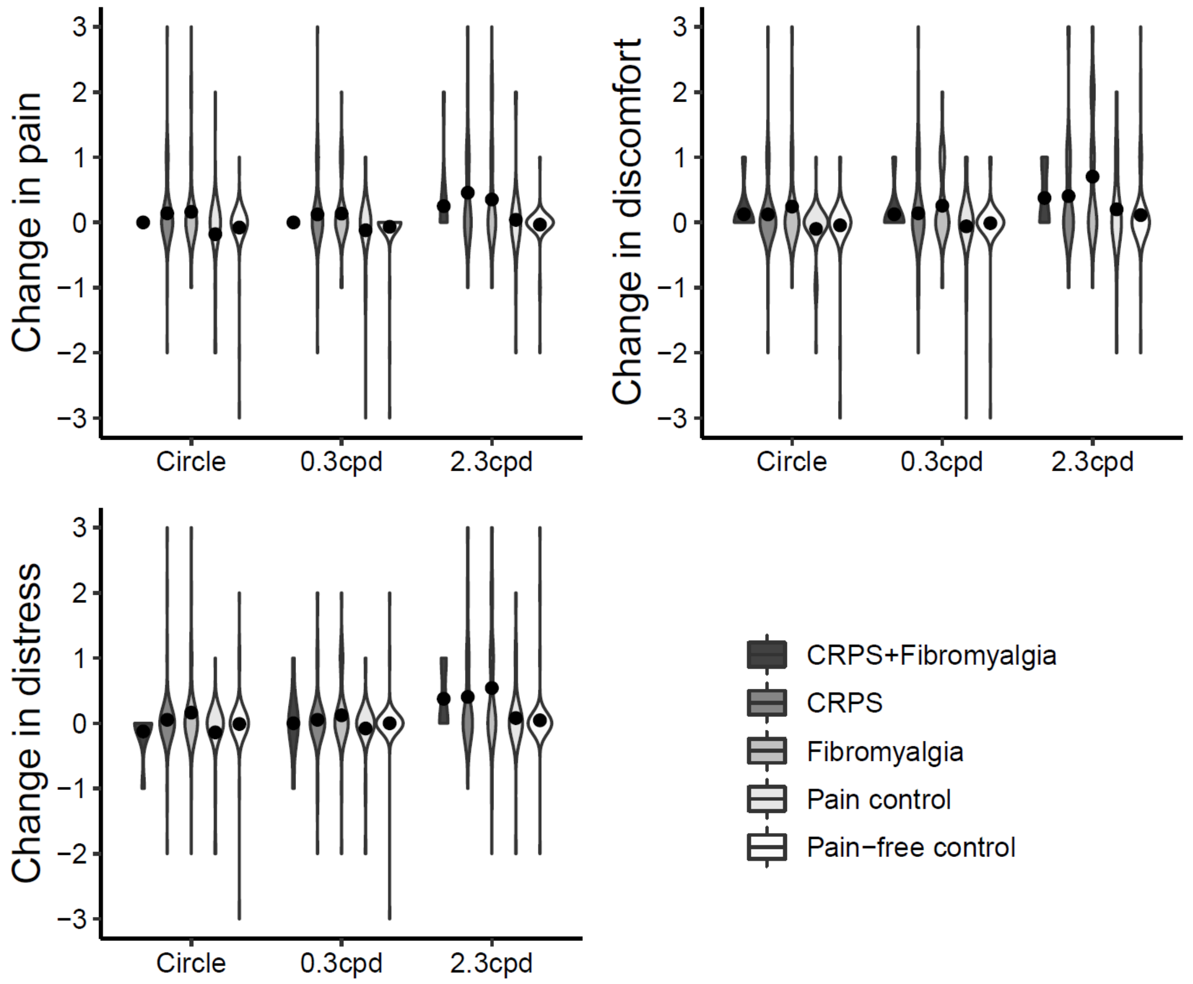

Supplementary Figure 2. Violin plots and mean scores (black dots) depicting the change in pain, discomfort, and distress for the grey circle, the 0.3cpd striped pattern, and the 2.3cdp striped pattern, on a scale from -3 (severe decrease) to +3 (severe increase), split for respondents with Complex Regional Pain Syndrome (CRPS) and fibromyalgia (*N* = 8), CRPS (*N* = 57), fibromyalgia (*N* = 74), pain controls (*N* = 50), and pain-free controls (*N* = 89). Exploratory Mann Whitney Tests (i.e. not corrected for multiple comparisons) showed that the CRPS+fibromyalgia group reported a larger increase in distress compared to the pain-free controls for the 2.3cpd stripes (U = 235.0, p < .001, r = -0.36). The CRPS+fibromyalgia group did not significantly differ from any of the other groups regarding changes in pain, discomfort, or distress for the circle and striped patterns (*ps* ≥ .132).

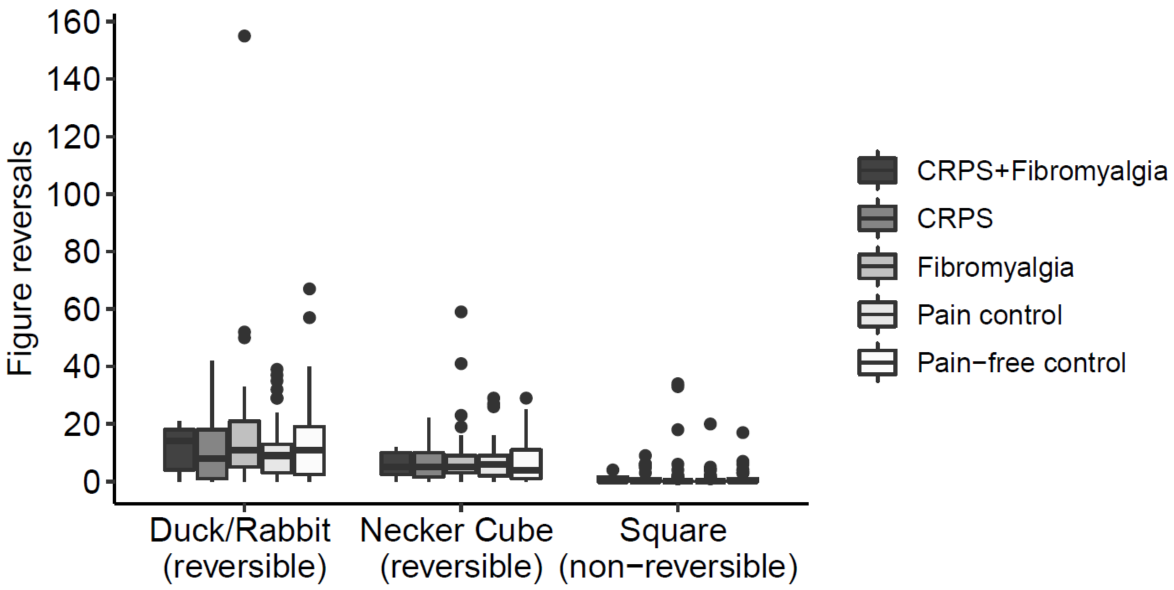

Supplementary Figure 3. Boxplots depicting the total number of reported figure reversals (measured by mouse clicks) for the reversible Duck/Rabbit figure, the reversible Necker Cube, and the non-reversible square, split for respondents with Complex Regional Pain Syndrome (CRPS) and fibromyalgia (N = 7), CRPS (N = 55), fibromyalgia (N = 73), pain controls (N = 49), and pain-free controls (N = 87). The thick line in the middle is the median. The top and bottom box lines show the first and third quartiles. The whiskers show the maximum and minimum values, with the exceptions of outliers (circles). Exploratory Mann Whitney Tests (i.e. not corrected for multiple comparisons) showed that the CRPS+fibromyalgia group did not significantly differ from any of the other groups regarding the number of reported figure reversals ($ps \geq .482$).

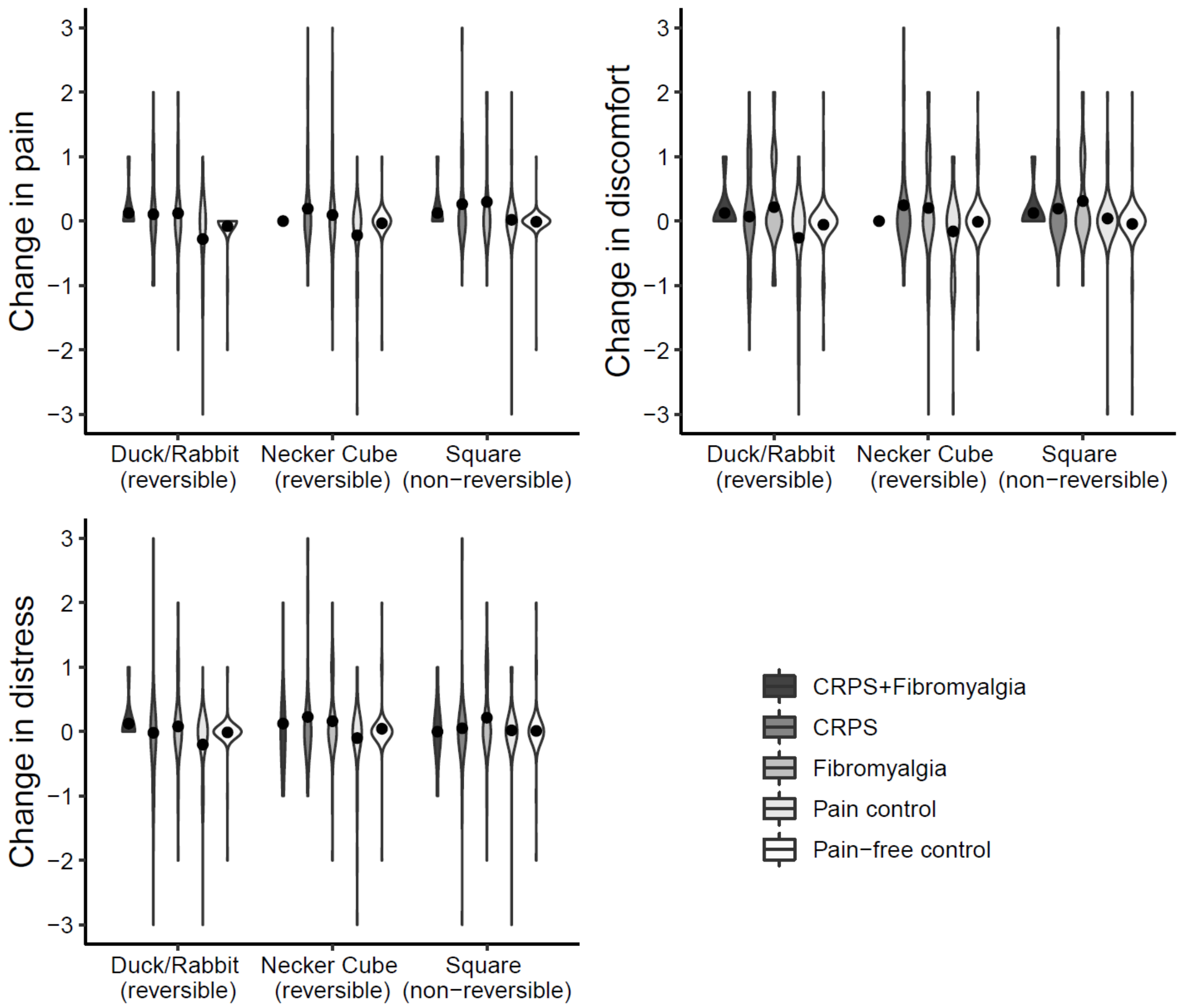

Supplementary Figure 4. Violin plots and mean scores (black dots) depicting the change in pain, discomfort, and distress for the reversible Duck/Rabbit figure, the reversible Necker Cube, and the non-reversible square , on a scale from -3 (severe decrease) to +3 (severe increase), split for respondents with Complex Regional Pain Syndrome (CRPS) and fibromyalgia (N = 8), CRPS (N = 57), fibromyalgia (N = 74), pain controls (N = 50), and pain-free controls (N = 89). Exploratory Mann Whitney Tests (i.e. not corrected for multiple comparisons) showed that the CRPS+fibromyalgia group did not significantly differ from any of the other groups regarding changes in pain, discomfort, or distress for the reversible images or the square ($ps \geq .051$).

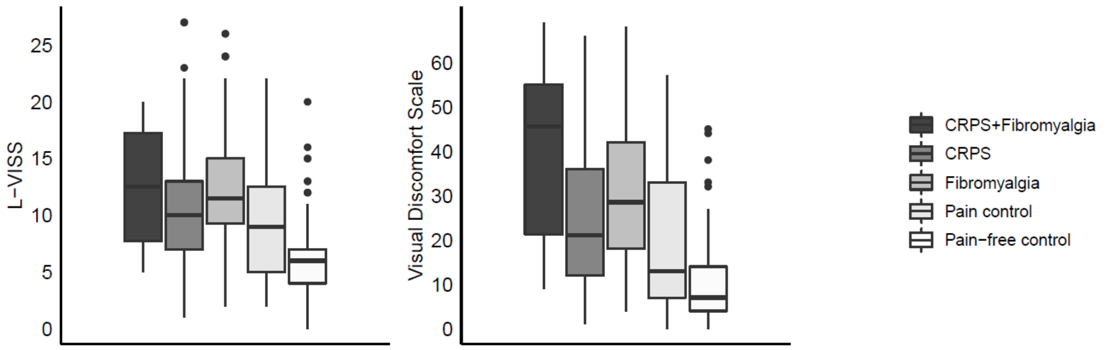

Supplementary Figure 5. Boxplots depicting the Leiden Visual Sensitivity Scale (L-VISS) and Visual Discomfort Scale, split for respondents with Complex Regional Pain Syndrome (CRPS) and fibromyalgia (N = 8), CRPS (N = 57), fibromyalgia (N = 74), pain controls (N = 50), and pain-free controls (N = 89). The thick line in the middle is the median. The top and bottom box lines show the first and third quartiles. The whiskers show the maximum and minimum values, with the exceptions of outliers (circles). Exploratory Mann Whitney Tests (i.e. not corrected for multiple comparisons) showed that the CRPS+fibromyalgia group reported more light and pattern sensitivity in daily life as measured with the VDS compared to the CRPS only group (U = 129.5, r = -0.24, p = .049), the other pain group (U = 77.5, r = -0.35, p = .008), and the pain-free group (U = 54.5, r = -0.40, p < .001). The CRPS+fibromyalgia group had higher scores on the L-VISS than the pain-free group (U = 115, r = -0.32, p = .001). The CRPS+fibromyalgia group did not significantly differ from any of the other groups regarding self-reported light and pattern sensitivity in daily life as measured with the L-VISS and/or VDS ($ps \geq$ .103).
